# Enhancing the Machinability of Sapphire via Ion Implantation and Laser-Assisted Diamond Machining

**DOI:** 10.3390/mi16101165

**Published:** 2025-10-14

**Authors:** Jinyang Ke, Honglei Mo, Ke Ling, Jianning Chu, Xiao Chen, Jianfeng Xu

**Affiliations:** 1 State Key Laboratory of Intelligent Manufacturing Equipment and Technology, School of Mechanical Science and Engineering, Huazhong University of Science and Technology, Wuhan 430074, China; jinyang_ke@hust.edu.cn (J.K.);; 2 Shanghai Aerospace Control Technology Institute, Shanghai 201109, China; 3 Hubei Key Laboratory of Modern Manufacturing Quality Engineering, School of Mechanical Engineering, Hubei University of Technology, Wuhan 430068, China

**Keywords:** sapphire, ion implantation, ultra-precision cutting, laser-assisted diamond machining

## Abstract

Sapphire crystals, owing to their outstanding mechanical and optical properties, which are widely used in advanced optics, microelectronic devices, and medical instruments. The manufacturing precision of sapphire optical components critically affects the performance of advanced optical systems. However, the extremely high hardness and low fracture toughness of sapphire make it a typical hard-to-machine material, prone to brittle surface fractures and subsurface damage during material removal. Improving the machinability of sapphire remains a pressing challenge in advanced manufacturing. In this study, surface modification and enhanced ductility of C-plane sapphire were achieved via ion implantation, and the machinability of the modified sapphire was further improved through laser-assisted diamond machining (LADM). Monte Carlo simulations were employed to investigate the interaction mechanisms between incident ions and the target material. Based on the simulation results, phosphorus ion implantation experiments were conducted, and transmission electron microscopy observation was used to characterize the microstructural evolution of the modified layer, while the optical properties of the samples before and after modification were analyzed. Finally, groove cutting experiments verified the enhancement in ductile machinability of the modified sapphire under LADM. At a laser power of 16 W, the ductile–brittle transition depth of the modified sapphire increased to 450.67 nm, representing a 51.57% improvement over conventional cutting. The findings of this study provide valuable insights for improving the ductile machining performance of hard and brittle materials.

## 1. Introduction

Sapphire possesses outstanding properties such as high strength, thermal stability, excellent transparency, and superior wear and impact resistance [1,2,3], making it an ideal substrate material for advanced optical systems in the fields of microelectronic devices and medical instruments [4,5,6]. However, the extremely high hardness and low fracture toughness of sapphire make it a typical hard-to-machine material with pronounced anisotropy [7,8]. Material removal during nanocutting of sapphire often leads to surface fracture and subsurface damage [9,10]. The ductile–brittle transition depth (DBTD) of sapphire generally falls within the range of 100–400 nm, with specific values strongly dependent on crystal orientation [7,11]. Such a small DBTD severely restricts the material removal rate and imposes stringent requirements on machine tool control, making it extremely challenging to achieve a consistent ductile regime cutting across the entire workpiece surface.

Single-point diamond turning (SPDT) is an important technology in ultra-precision manufacturing for fabricating complex optical surfaces. It offers advantages of high precision, high efficiency, and excellent machining flexibility, and has attracted widespread attention over the past few decades [12,13]. Nevertheless, the extreme hardness of sapphire often causes severe tool wear, leading to a dramatic reduction in tool life [11,14,15]. In addition, subsurface damage introduced during machining can deteriorate optical path accuracy and compromise the service performance of optical systems [16]. Currently, the traditional processing route for sapphire optical components involves grinding, lapping and polishing. However, strong grinding forces result in poor surface quality, and the inevitable subsurface damage layer requires complex post-treatment [10,17,18]. Moreover, tool–workpiece interference and edge effects during grinding and polishing hinder the achievement of high formation accuracy [17]. These limitations hinder conventional processes from achieving high-precision and low-damage manufacturing of hard and brittle optical components. They also underscore the urgent need for technological advances beyond traditional methods.

**Figure 1:**
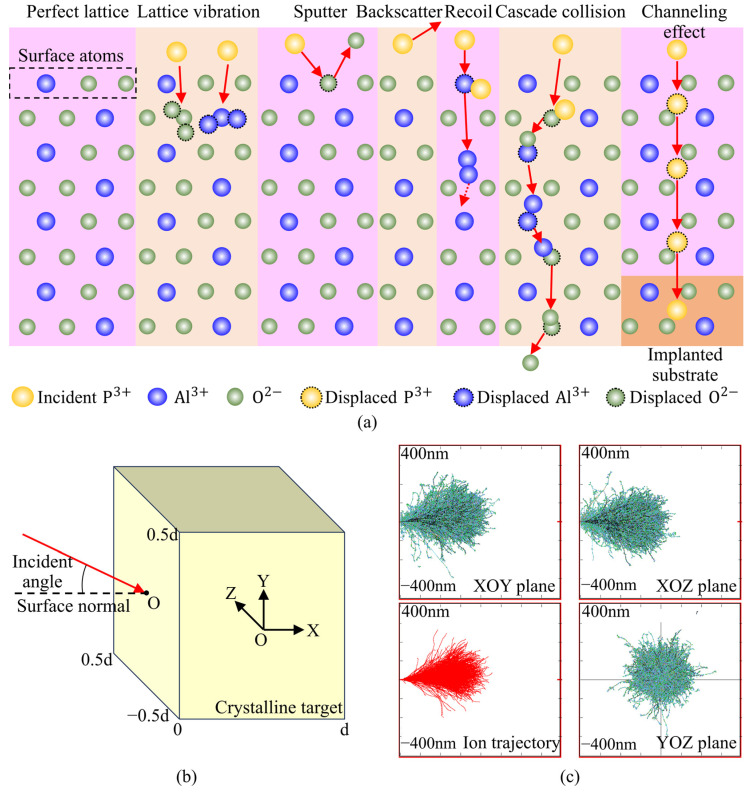
Simulation of energetic ion implantation in sapphire crystal. (**a**) Schematic illustration of the ion implantation process where the red arrows indicate the direction of atomic/ionic motion; (**b**) three-dimensional model used for TRIM simulation; (**c**) snapshot of P ions with energy of 400 keV at an incidence angle of 7°.

Ion implantation offers a promising strategy for tailoring the mechanical properties and crystal structure of materials, thereby reducing the hardness and brittleness of sapphire. During ion implantation, interactions between high-energy particles and the target material can trigger a variety of important physical processes, including sputtering, backscattering, and the formation of vacancies and interstitial atoms, as illustrated in Figure 1a. Irradiation of sapphire by energetic ions triggers complex collision events. High-energy ions exceeding the displacement threshold displace lattice atoms, generating vacancies, initiating cascades, or causing sputtering. Low-energy ions below the threshold mainly induce lattice vibrations and dissipate energy thermally without permanent damage. In some cases, incident ions may channel through the crystal, forming isolated vacancies at the ends of their trajectories [19,20]. Skuratov et al. [21] investigated four crystallographic orientations of sapphire crystals irradiated with Kr (305 MeV), Xe (595 MeV), and Bi ions (710, 557, 269, and 151 MeV). They reported that obvious surface defects could only be observed under Bi ion irradiation with energies higher than 269 MeV. Kabir et al. [22] conducted irradiation experiments on sapphire crystals using 0.7 MeV Xe ions at doses ranging from 5 × 10^11^ to 2 × 10^14^ ions/cm^2^. Their results showed that when the dose exceeded 1.2 × 10^13^ ions/cm^2^, the crystal structure became completely disordered starting from the surface. Current research on sapphire modification has mainly focused on defect formation mechanisms, while systematic investigations into the influence of implantation parameters on ion–target interactions remain limited.

High-energy ion implantation can generate various damage structures, ranging from isolated point defects and defect clusters to the formation of amorphous regions [23]. Such damage can reduce the hardness and brittleness of crystals, thereby enabling enhanced plastic deformation during material removal [24]. Fang et al. [19] proposed a novel method termed “nanometric machining of ion-implanted materials” in which fluorine ions were implanted into single-crystal silicon to form a 4.5 μm thick amorphous modified layer, followed by diamond cutting for ductile regime material removal. Their results showed that the DBTD increased to 923.566 nm, the surface roughness Ra reached 0.861 nm, and the tool life was extended by five times. Wang et al. [25] investigated the modification effects of oxygen ion implantation on the nanocutting of single-crystal silicon. Their experimental results showed that the irradiation-induced damage absorbed shear strain energy during the nanocutting process, thereby promoting plastic deformation of the material. To et al. [26] modeled and visualized the distribution of implanted hydrogen ions and the displacement damage induced in the subsurface of single-crystal silicon, and further validated the enhanced machinability of surface-modified silicon through ultra-precision micro-cutting experiments. However, the hardness of silicon is much lower than that of sapphire, highlighting the urgent need to further explore the potential of ion implantation surface modification in improving ductile regime machining of hard and brittle materials.

Over the past decades, laser-assisted machining (LAM) has demonstrated great potential in processing hard and brittle materials. For metals such as titanium alloys and superalloys, LAM has been shown to effectively reduce yield strength, thereby lowering cutting forces [27], improving surface integrity [28], and extending tool life [29]. Mohammadi et al. [30] proposed laser-assisted diamond machining (LADM), wherein a focused laser beam is transmitted through a transparent tool to irradiate the cutting zone directly, enabling localized and precise heating to enhance the machinability of hard and brittle materials. Numerous experimental studies have verified the effectiveness of LADM for the ultra-precision cutting of silicon [31], silicon carbide [32], and tungsten carbide [33,34]. Reported benefits include reduced cutting forces and tool wear, suppression of surface and subsurface defects, improved machining quality, and enhanced processing efficiency [32,34,35,36]. However, given the high transmittance of sapphire over a broad spectral range, the effectiveness of LADM of sapphire remains uncertain.

To address the poor machinability of sapphire in ultra-precision cutting, this study employed ion implantation to achieve surface modification and enhance the ductility of C-plane sapphire, followed by the application of LADM to further improve ductile machinability. Monte Carlo simulations were conducted to investigate the ion–target interaction mechanisms during implantation. Based on the simulation results, phosphorus ion implantation experiments were performed, and transmission electron microscopy (TEM) was employed to characterize the microstructural evolution of the ion-induced modified layer. The optical properties of sapphire samples before and after implantation were also analyzed. Finally, groove cutting experiments were conducted to validate the enhancement of the machinability of the modified sapphire under LADM.

## 2. Materials and Methods

### 2.1. Basic Principles and Simulation of Ion Implantation

The ion–target interaction process is accompanied by complex energy exchange mechanisms. The displacement energy, $E_{disp}$, refers to the minimum energy required to displace a target atom sufficiently far from its lattice site so that it cannot quickly return. The lattice binding energy, $E_{latt}$, is defined as the minimum energy required to remove an atom completely from the lattice. The surface binding energy, $E_{surf}$, refers to the energy needed to remove an atom from the surface of the target material. Atoms with a final energy, $E_{final}$, below the threshold are considered stationary within the crystal target. When a moving atom collides with a target atom and transfers energy exceeding $E_{disp}$, the target atom is displaced from its lattice site. As it loses $E_{latt}$ to the lattice, its recoil energy is given by $E_{recoil}$ = $E_{disp}$−$E_{latt}$. If the recoiled target atom possesses energy greater than $E_{disp}$, it can continue to collide with other target atoms, generating additional vacancies, i.e., a collision cascade. For sapphire crystal targets, the default values for aluminum atoms are: $E_{disp}$ = 25 eV, $E_{latt}$ = 3 eV, and $E_{surf}$ = 3.36 eV; for oxygen atoms: $E_{disp}$ = 28 eV, $E_{latt}$ = 3 eV, and $E_{surf}$ = 2 eV [37,38].

In this study, the TRIM (Transport of Ions in Matter) Monte Carlo simulator from the SRIM-2013 package, developed by J.F. Ziegler and J.P. Biersack, was employed to calculate the collision processes between high-energy ions and the target material [39]. The simulator describes ion–target collisions using the Ziegler–Biersack–Littmark (ZBL) universal potential [40] and establishes a complete theoretical framework based on extensive experimental data. Figure 1b shows the three-dimensional model and coordinate system used for the TRIM simulations. The target material was modeled as a cube with a length of *d* = 800 nm, and energetic phosphorus (P) ions were obliquely incident on the target surface with energies ranging from 100 to 600 keV. TRIM features a graphical user interface and a comprehensive compound target database, allowing the simulation of various physical processes, including target damage, ion ranges, backscattering yields, sputtering yields, ionization, and phonon generation [41]. Each simulation tracked 1000 ions to ensure statistical reliability. The “detailed full-cascade” mode was employed to simulate the ion implantation experiments, providing data on backscattering yields, vacancy numbers, ion range distributions, and energy exchange processes. All ion trajectories were traced using the binary collision approximation. Figure 1c presents a snapshot of P ions with an energy of 400 keV at an incidence angle of 7° at the end of the implantation process. The primary source of energy loss arises from nuclear stopping, while incident ions continuously and uniformly lose energy through interactions with target electrons in the intervals between collisions.

### 2.2. Subsurface Structural Characterization

The experimental specimens were procured from TDG Holding Co., Ltd., (Haining, China), with a diameter of 34 mm and a thickness of 3 mm. It should be noted that the selected implantation dose in this study ensures sufficient amorphization of the substrate over a larger depth range, as theoretically calculated in our previously published study [42]. TEM samples were extracted from specific regions of grooves obtained in groove cutting experiments using a focused ion beam (FIB) system (Helios Nanolab 660, FEI Company, Hillsboro, OR, USA). Prior to preparation, the workpieces were mounted on conductive tape and sputter-coated with gold for 240 s to enhance conductivity, followed by deposition of a tungsten protective layer to prevent potential damage during FIB processing. Aberration-corrected TEM (Titan G2 60–300 probe, FEI Company, Hillsboro, OR, USA) was employed to examine the sample cross-sections and perform selected-area electron diffraction (SAED) analysis, enabling characterization of subsurface microstructural changes induced by ion implantation.

### 2.3. Groove Cutting Tests

**Figure 2:**
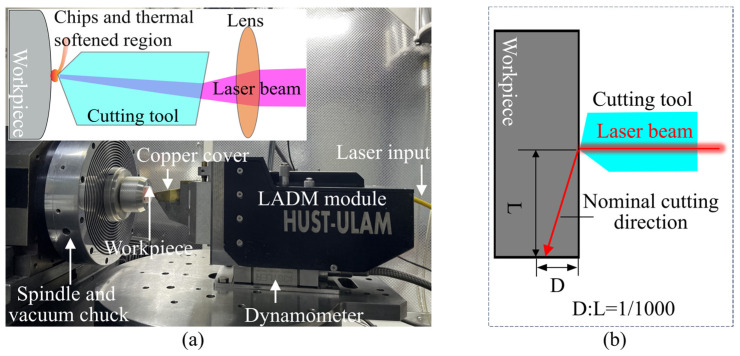
Experimental validation of LADM cutting. (**a**) Photograph of the experimental setup; (**b**) schematic illustration of the groove cutting experiment.

In LADM, a focused laser beam passes through a transparent tool and directly irradiates the cutting edge to locally and precisely heat the material, as shown in Figure 2a. This localized laser irradiation reduces the cutting resistance of brittle and hard-to-machine materials through thermal softening, promotes shear deformation, and facilitates chip formation along the rake face. The light source in LADM is a continuous 1070 nm fiber laser. In the experiments, the laser spot was precisely aligned with the tool tip, and the output power at the cutting edge was measured using a power meter (30 (150) A-BB-18, Ophir, Jerusalem, Israel). A custom in situ laser-assisted diamond tool with a −35° rake angle and a 0.506 mm tool tip radius was employed for constant-speed lateral feed. During machining, the cutting depth of each groove was continuously increased from zero to the maximum, as illustrated in Figure 2b. Based on the maximum *X*-axis travel and the dynamic response of the feed system, a cutting speed of 200 mm/min was chosen to ensure optimal machine performance. Each experiment was repeated three times to ensure data reliability. In the self-developed LADM setup, the maximum stable output power measured at the diamond tool cutting edge was 20 W. To prevent high-temperature failure of the laser and ensure stable operation of the cutting system, the maximum power applied in the groove cutting experiments was set to 16 W. The parameters of the groove cutting experiments are listed in Table 1. It is worth noting that in this study, the cutting speed was kept constant while the laser power was set at five evenly spaced levels, in order to intuitively demonstrate the enhancement of ductile cutting performance of modified sapphire under LADM.

### 2.4. Characterization Method

The grooves obtained from groove cutting experiments with varying cutting depths were observed using an optical microscope (DSX 510, Olympus Corporation, Tokyo, Japan) to examine their surface morphology. The DBTD of the grooves was measured using a white-light interferometer (NewView 9000, ZYGO Corporation, Middlefield, CT, USA). DBTD was defined as the depth at which the first crack appeared on the machined surface. The chips generated during machining were collected and examined using a scanning electron microscope (GeminiSEM 360, ZEISS Group, Oberkochen, Baden-Württemberg, Germany).

## 3. Results and Discussions

### 3.1. Effect of Energy on Ion–Target Interactions

**Figure 3:**
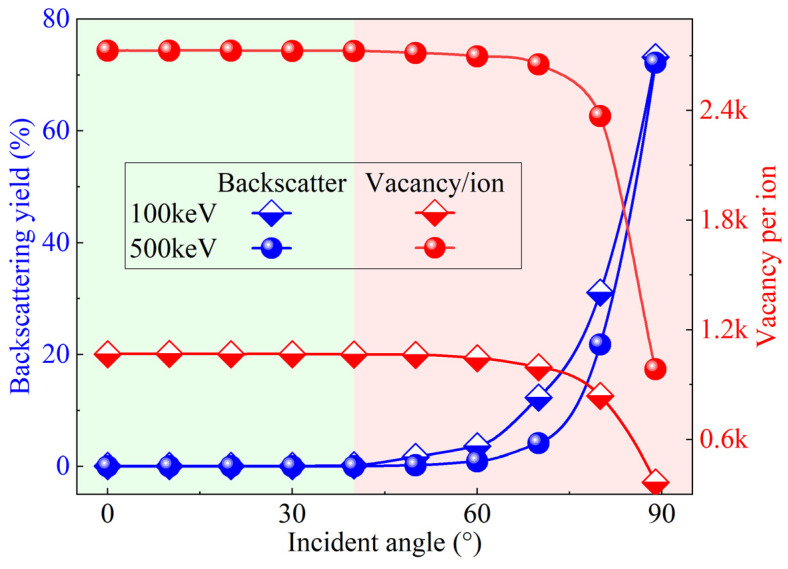
Effect of incident angle on implantation behavior at different incident energies.

Figure 3 illustrates the effect of the incidence angle on the implantation behavior of P ions in sapphire crystals. First, as the incident energy increases from 100 keV to 500 keV, the backscattering fraction gradually increases with increasing incidence angle. In contrast, the vacancy-to-ion ratio exhibits the opposite trend, decreasing progressively with increasing incidence angle. Notably, when the incidence angle is less than 40°, both parameters remain relatively stable. The backscattering fraction stays near zero, while the vacancy-to-ion ratio reaches about 1000 at 100 keV and 2700 at 500 keV. When the incidence angle exceeds 40°, the backscattering fraction increases exponentially with the angle. This observation indicates that ion implantation is more stable under small-angle incidence conditions. In engineering practice, the incidence angle is typically set to 7° to avoid channeling effects and ensure uniform implantation. Accordingly, a fixed incidence angle of 7° was used in both the simulations and experiments of this study. This approach not only minimizes ion scattering losses but also ensures a sufficient defect generation rate within the modified layer.

**Figure 4:**
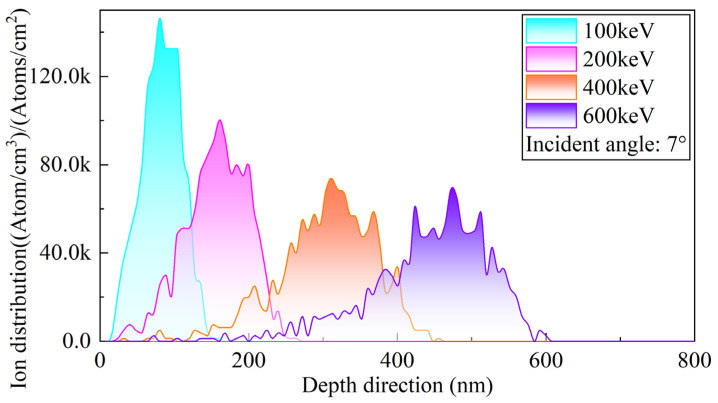
Depth distributions of P ions at different incident energies with an incidence angle of 7°.

Figure 4 shows the depth profiles of P ions implanted at different incident energies under a fixed incidence angle of 7°. It can be observed that, as the incident energy increases, the peak positions of the P ion distributions gradually shift deeper into the target, indicating that the average penetration depth of the ions increases with energy. At the same time, the shape of the ion distribution curves changes. The peak concentration decreases, while the distribution broadens. This indicates that high-energy ions undergo a longer energy dissipation process within the target, resulting in a more dispersed spatial distribution of implanted ions. This behavior reflects a fundamental phenomenon in ion implantation: under the same material and incidence angle conditions, the projected range of ions increases nonlinearly with incident energy, while the local peak concentration is diluted due to the extended penetration depth. It is noteworthy that the unit of ion concentration is expressed as (Atoms/cm^3^)/(Atoms/cm^2^), representing the probability density of ion distribution per unit implantation dose. Although this unit does not directly correspond to the absolute concentration, multiplying it by the actual implantation dose (in ions/cm^2^) yields the true dopant concentration (in Atoms/cm^3^). Therefore, these results provide a theoretical basis for selecting appropriate implantation energy and dose combinations to achieve a desired dopant depth and concentration.

**Figure 5:**
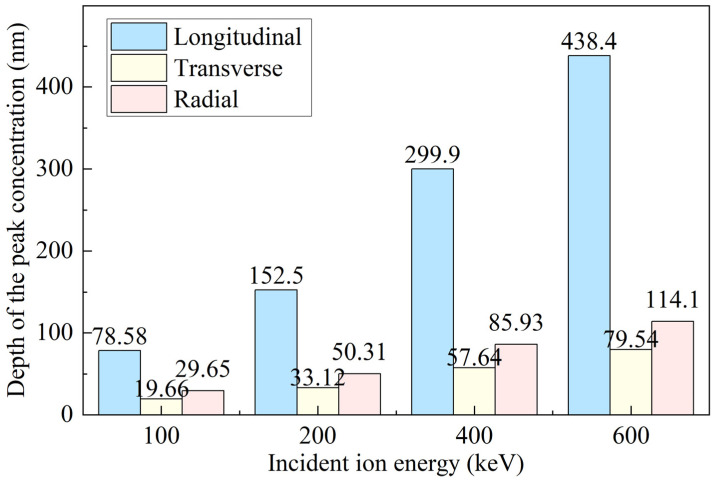
Statistical analysis of the peak concentration depths of P ions.

To investigate the three-dimensional migration behavior of ions, Figure 5 analyzes the projected ranges of P ions in the three orthogonal planes (XOY, XOZ, and YOZ), corresponding to the depths at which peak concentrations occur. It is evident that, as the ion energy increases, the projected ranges in all three directions increase significantly. This is because high-energy ions travel longer paths within the target, undergo more collisions, and thus exhibit a broader spatial distribution. Comparing different directions, the longitudinal projection (within the XOY plane) remains significantly greater than the transverse (XOZ) and radial (YOZ) projections. This difference arises from the fact that ions predominantly propagate along the incidence direction with limited scattering angles, resulting in a penetration depth along the normal that is much greater than the lateral spread within the plane. In addition, the 7° oblique incidence induces a slight lateral shift, causing a certain asymmetry in the ion distribution within the transverse plane, as shown in Figure 1c.

**Figure 6:**
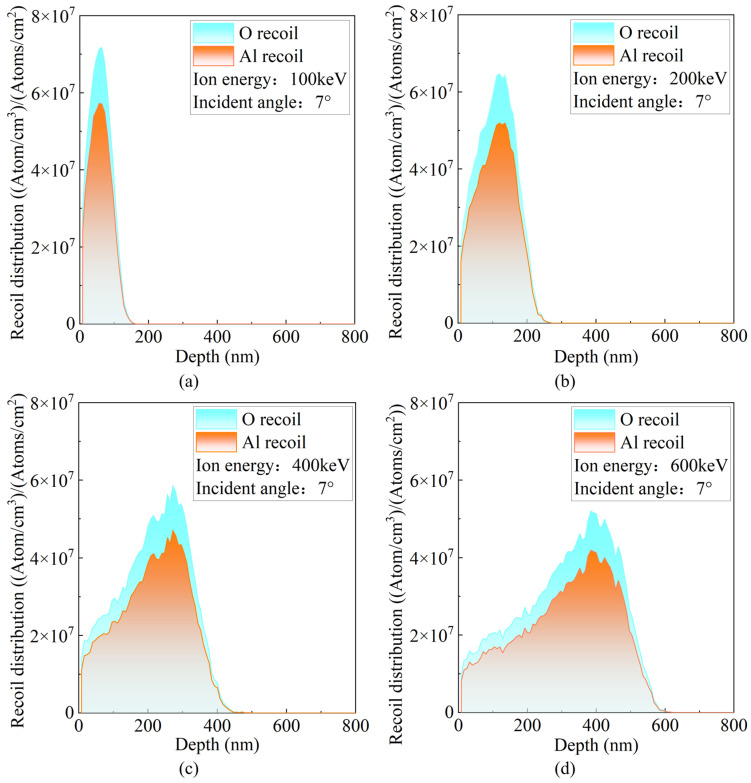
Recoil distributions of target atoms at different incident energies. (**a**) 100 keV; (**b**) 200 keV; (**c**) 400 keV; (**d**) 600 keV.

The regulation of recoil atom behavior by the incident ion energy is a key physical mechanism for achieving ordered microstructural evolution and constructing an ideal modified state in materials. Figure 6 illustrates the distributions of recoiled target atoms induced by P ions at different incident energies. As the ion energy increases, the overall density of recoiled atoms decreases slightly. However, their extension along the depth direction becomes more pronounced, resulting in a marked broadening of the spatial distribution. This trend indicates that high-energy ions can trigger deeper atomic collision events, causing more profound perturbations to the underlying crystal structure and potentially altering the macroscopic mechanical properties of the material. Regarding the composition of recoiled atoms, the density of oxygen recoils is consistently higher than that of aluminum. This can be explained from the perspectives of crystal structure and atomic mass. On one hand, oxygen atoms constitute a higher proportion of the sapphire lattice, making them more likely to undergo recoil events during collisions. On the other hand, because oxygen atoms are lighter and have a lattice binding energy comparable to aluminum, they are more easily displaced by ion collisions. In contrast, the heavier aluminum atoms tend to remain stable.

**Figure 7:**
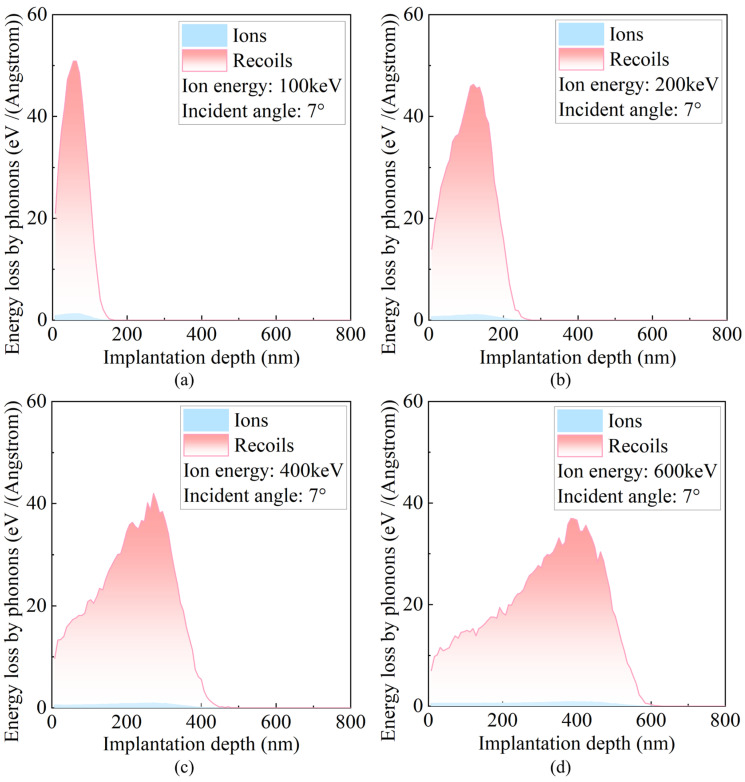
Energy distributions transferred via phonons by incident ions and recoiled target atoms at different incident energies. (**a**) 100 keV; (**b**) 200 keV; (**c**) 400 keV; (**d**) 600 keV.

When ions or recoiled atoms with different kinetic energies collide within a crystal, their effects on target atoms depend on whether the transferred energy exceeds the displacement energy $E_{disp}$. If the energy transferred during a collision is below this threshold, the target atom may undergo slight displacement or vibration, but not enough to leave its original lattice site. Such collisions do not create permanent defects; instead, they induce local lattice perturbations, manifesting as short-lived oscillations of atoms around their equilibrium positions. In this process, the kinetic energy is no longer propagated as momentum but is converted into phonons via lattice vibrations. Due to strong coupling between atoms in the crystal, the vibration of one atom induces synchronous vibrations in neighboring atoms, generating phonons that propagate as waves. When an atom is displaced from its original lattice site, its binding energy is transferred to phonons generated by the recoiled atom. The remaining phonons are produced by ions or recoiled atoms that transfer energies below $E_{disp}$ to lattice atoms. As shown in Figure 7, at low ion energies, recoiled atoms are mainly concentrated near the surface, and the corresponding phonon energy deposition is similarly localized. With increasing ion energy, recoiled atoms can penetrate deeper into the lattice, triggering collisions and causing the spatial distribution of phonons to extend further into the material. The peak depth of phonon energy deposition shifts deeper with increasing ion energy, indicating that the region of active atomic vibrations within the material also moves inward. This is because high-energy ions are more likely to induce oscillations of deeper atoms. Simultaneously, the peak intensity of phonon energy deposition slightly decreases, suggesting that the number of locally excited phonons per unit volume is reduced. In other words, energy is distributed more uniformly, and local accumulation effects are diminished. Further analysis reveals that incident ions themselves contribute negligibly to phonon generation, which is almost entirely produced by recoiled target atoms.

**Figure 8:**
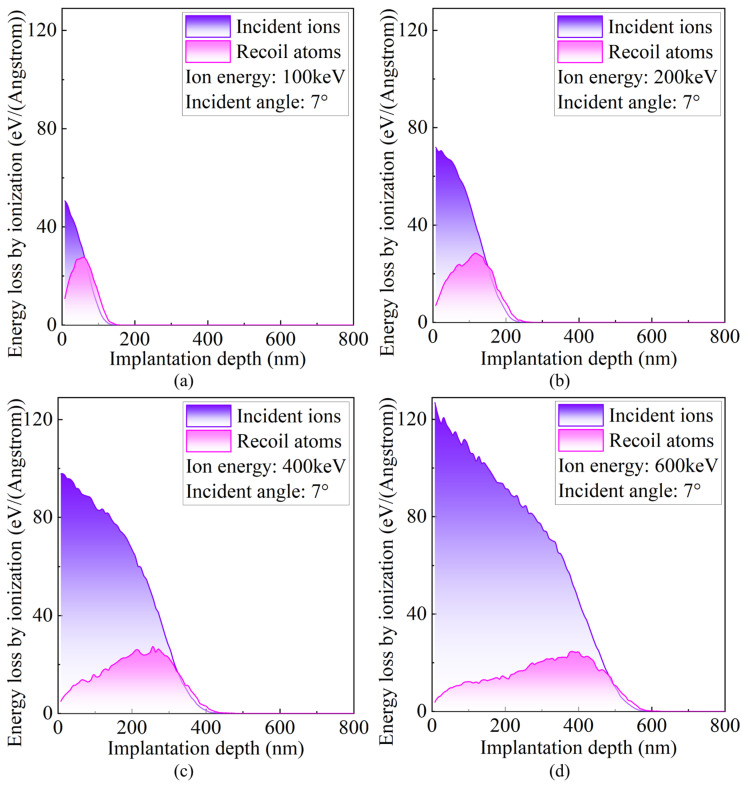
Energy distributions transferred via ionization by incident ions and recoiled target atoms at different incident energies. (**a**) 100 keV; (**b**) 200 keV; (**c**) 400 keV; (**d**) 600 keV.

Figure 8 presents the energy distribution transferred via ionization from incident ions and recoiled target atoms at different incident energies. Ionization energy loss refers to the energy dissipated by ions due to interactions with the target’s valence electrons during their motion, representing another major mechanism of energy dissipation in ion–material interactions. As P ions penetrate the crystal and interact with valence electrons, the extent of energy loss depends on the stopping power of these electrons. The simulation results show that, with increasing incident energy, the ionization energy losses induced by both incident ions and recoiled target atoms increase. This trend reflects that high-energy particles possess longer trajectories and higher kinetic energies within the crystal, enabling more inelastic collisions with electrons and resulting in more extensive and intense electronic excitation. Moreover, the ionization energy loss of incident ions is consistently higher than that of recoiled atoms, primarily due to the velocity difference between the two. Although both phonon and ionization energy dissipation mechanisms do not directly generate structural defects, their excitation intensity and spatial distribution significantly influence the thermal response, stress relaxation mechanisms, and local temperature rise during ion implantation. These effects provide important guidance for the optimization of ion implantation process parameters.

**Figure 9:**
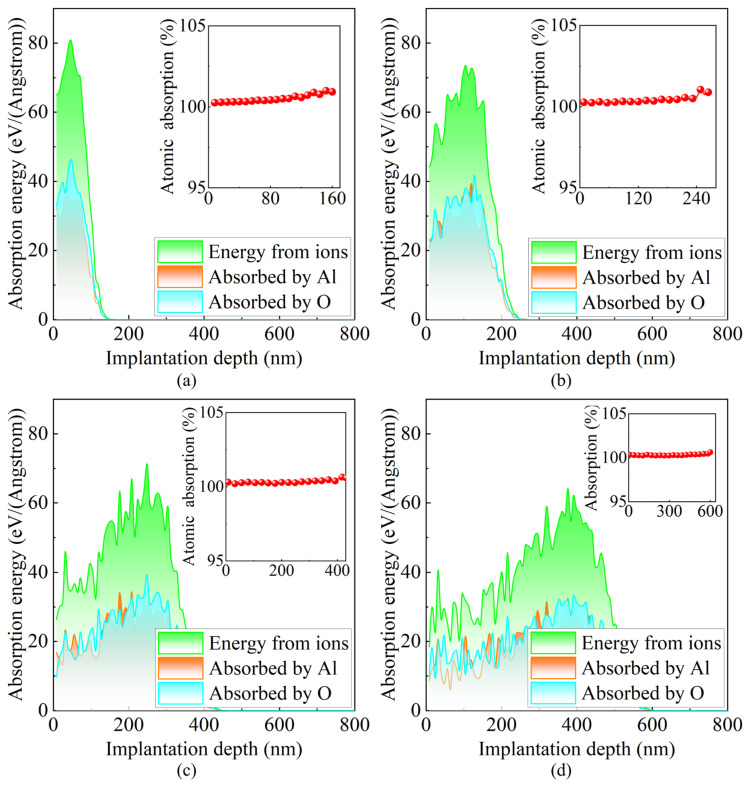
Distributions of deposited ion energy and energy absorbed by target atoms at different incident energies. (**a**) 100 keV; (**b**) 200 keV; (**c**) 400 keV; (**d**) 600 keV. The red dashed curve in the inset shows the ratio of the total energy absorbed by oxygen and aluminum atoms to the total deposited ion energy.

Figure 9 illustrates the distributions of total deposited ion energy and energy absorbed by target atoms at different incident energies. As the incident energy increases from 100 keV to 600 keV, the peak depth of energy deposition shifts significantly deeper into the material, and the distribution range broadens. This trend indicates that high-energy ions possess greater penetration capability and a wider energy release zone, with energy no longer confined to the near-surface region but gradually diffusing deeper into the material, thereby affecting a larger volume of the crystal structure. Within the main region of energy deposition, corresponding to the average ion range, the energy absorbed by target atoms changes relatively smoothly, suggesting that incident ions continuously transfer energy to lattice atoms during deceleration. Near the stopping end of the ions, the kinetic energy is rapidly exhausted, resulting in a sharp decrease in transferred energy. Under all implantation conditions, the energies absorbed by oxygen and aluminum atoms are comparable, showing no significant bias. To evaluate the utilization efficiency of deposited ion energy, the ratio of the total energy absorbed by oxygen and aluminum atoms to the total deposited ion energy was quantified. The results indicate that this ratio approaches 100% for all incident energies, meaning that nearly all deposited energy is ultimately absorbed by target atoms. From the perspective of material modification, this highly localized and efficient energy absorption process facilitates precise control over the modified layer structure, enabling manipulation of surface stress states, induction of crystal phase transformations, or suppression of crack propagation, and thus serves as a critical mechanism for achieving controllable microstructural evolution.

**Figure 10:**
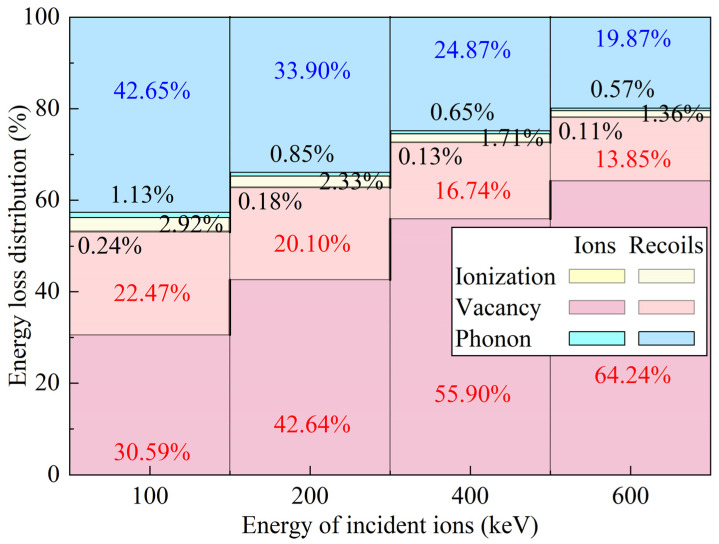
Energy dissipation distributions during the ion implantation process.

Based on the energy distributions of ions and recoiled atoms described above, the energy dissipation pathways and proportions during ion implantation were further analyzed, as shown in Figure 10. At different incident energies, at least 53% (up to 78.09%) of the total energy of incident particles is consumed through vacancy formation, thereby inducing defects within the crystal target, while at least 20% (up to 43.78%) is ultimately dissipated as phonons. This indicates that phonon dissipation plays a dominant role in ion implantation and represents the primary mechanism for converting kinetic energy into internal energy. Further analysis shows that, with increasing incident energy, the energy transferred to phonons by recoiled atoms decreases, whereas the energy transferred by incident ions gradually increases. These results demonstrate that varying the incident energy significantly affects the dominant phonon energy transfer mechanism. High-energy phonons generated by atomic collisions lead to local temperature rises, which subsequently diffuse to surrounding lower-temperature regions, enabling outward energy transfer.

**Figure 11:**
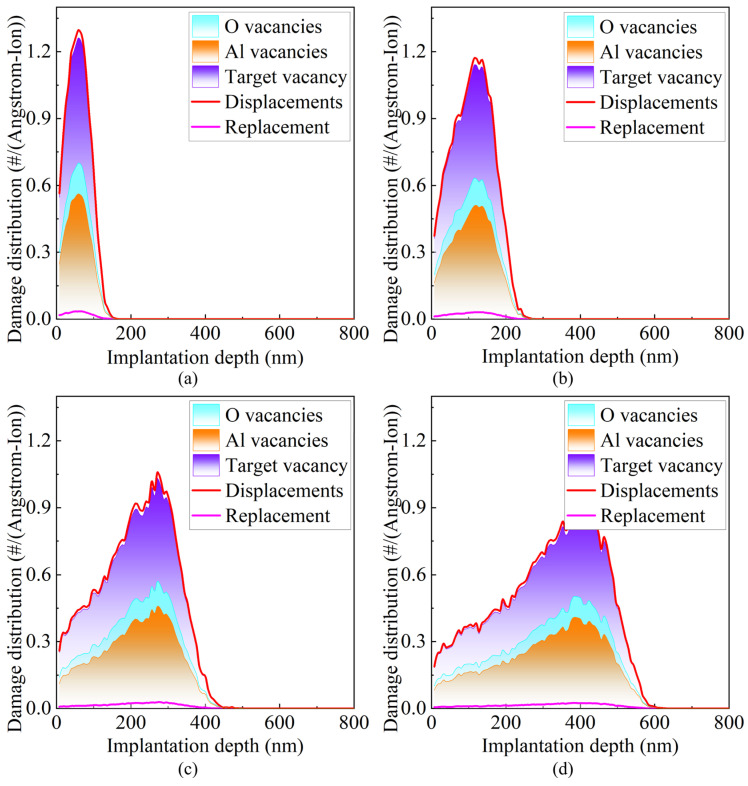
Spatial distributions of various types of damage formed in the target under different incident energies. (**a**) 100 keV; (**b**) 200 keV; (**c**) 400 keV; (**d**) 600 keV.

**Figure 12:**
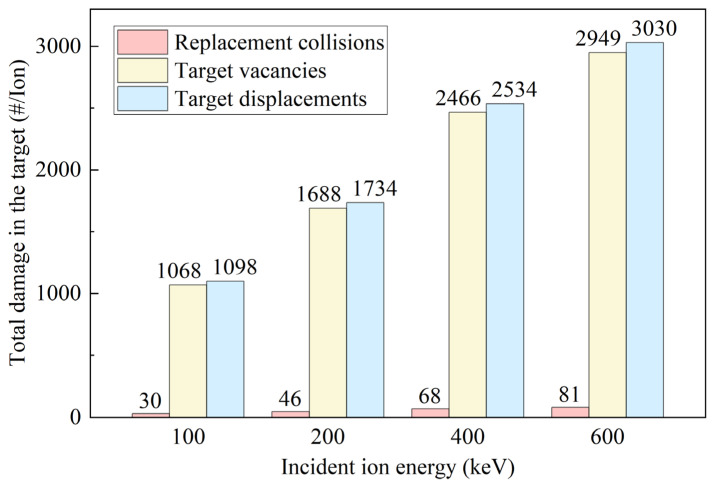
Statistical analysis of the total number of displacements, vacancies, and replacement collisions at different incident energies.

Figure 11 shows the spatial distributions of various types of damage, including vacancies, displacements, and replacement collisions, formed in the target material under different incident energies. It can be observed that, as the incident ion energy increases, the spatial extent of all damage types gradually expands, indicating that the damage penetration depth increases with energy. Meanwhile, the peak intensity decreases, suggesting that the damaged energy is spread over a larger volume, reducing local concentration. Notably, the combined intensity of oxygen and aluminum vacancies equals the total vacancy intensity of target atoms. In comparison, the overall vacancy curve lies below the corresponding displacement curve, indicating that the number of vacancies is smaller than the number of displacements, and not all displacement events produce stable vacancies. The bottommost curve represents the distribution of replacement collisions, which occur when incident atoms lose almost all their energy and can no longer advance, filling the sites left by recoiled target atoms. In other words, an incident atom displaces a target atom and then occupies its lattice site. Since they are the same element, no net change occurs in the target. Based on the damage distribution data in Figure 11, the total damage in the target at different incident energies was quantified by integration, as shown in Figure 12. It is noteworthy that, under all conditions, the total number of displacements equals the sum of vacancies and replacement collisions.

### 3.2. Microstructural Evolution of the Modification Layer

**Figure 13:**
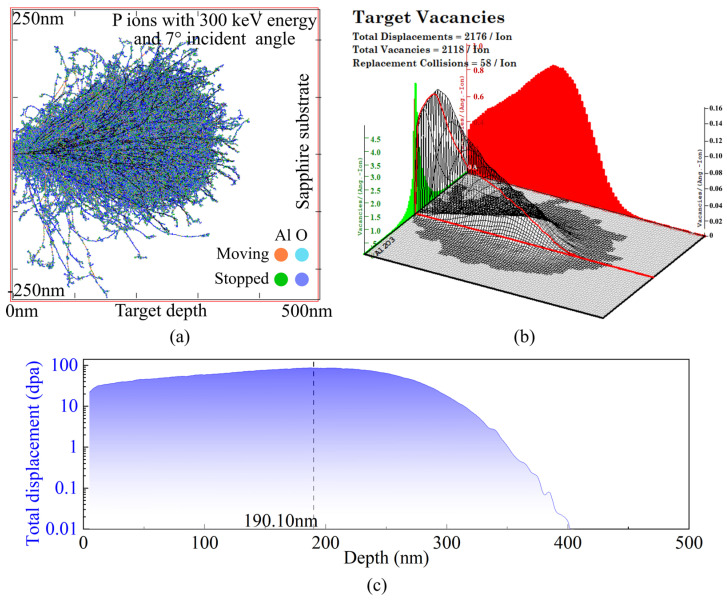
TRIM simulation results of 300 keV phosphorus ion implantation into a sapphire crystal at an incident angle of 7°. (**a**) Visualization of recoil cascade processes and ion trajectories; (**b**) three-dimensional distribution of target vacancies; (**c**) depth profile of total displacement damage.

Based on the Monte Carlo simulation results, phosphorus ions were implanted into C-plane single-crystal sapphire at room temperature using a medium-beam ion implanter (CI-S300MS) developed by China Electronics Technology Group Corporation (Beijing, China), with an incidence angle of 7°. To balance time efficiency and equipment performance, an implantation energy of 300 keV was selected. To ensure the formation of a homogeneous and stable modification layer, the ion implantation dose (i.e., number of ions per unit area) must be carefully optimized. Figure 13a illustrates, using different colors, the spatial distribution of recoil cascades and ion trajectories. The incident ions generate continuous lattice damage, while the cascades triggered by recoiled target atoms result in the formation of defect clusters. Figure 13b depicts the vacancy intensity profile in sapphire subjected to 300 keV phosphorus ion implantation. $A_{vacancy}$ denotes the mean number of vacancies created per unit ion range. Multiplying this value by the implantation dose yields the vacancy concentration per unit volume, expressed as follows:

(1) $$D_{vacancy}=\;10^{8}\times A_{vacancy}\times n_{dose},$$

Here, $A_{vacancy}$ is obtained from TRIM simulations, and $n_{dose}$ represents the implantation dose. Once $D_{vacancy}$ exceeds the atomic density of sapphire (1.175 × $10^{23}$ atoms/cm^3^), the material undergoes an amorphization transition. As shown in Figure 13b, the peak vacancy intensity along depth reaches ~1.01, implying that amorphization at a depth of 190.10 nm requires a minimum implantation dose of 1.163 × $10^{15}$ ions/cm^2^. However, since the vacancy intensity near the surface is only ~0.26, a much higher dose would be necessary to achieve full amorphization across the entire 400 nm depth. To guarantee sufficient amorphization of the substrate over a broader depth range, this study adopts 1% of the peak vacancy intensity as a reference, ultimately determining the implantation dose to be 1 × 10^17^ ions/cm^2^. Considering both the implantation dose and the atomic density of sapphire, the following equation presents the calculation method for displacement damage *ω* (displacements per atom, dpa) derived from TRIM output [43]:

(2) $$𝜔\;=\;A_{vacancy}\frac{10^{8}n_{dose}}{\rho },$$

where *ρ* is the atomic density of sapphire. Figure 13c shows that the displacement damage extends over a depth of ~400 nm, reaching a maximum of 86.16 dpa at 190.10 nm.

**Figure 14:**
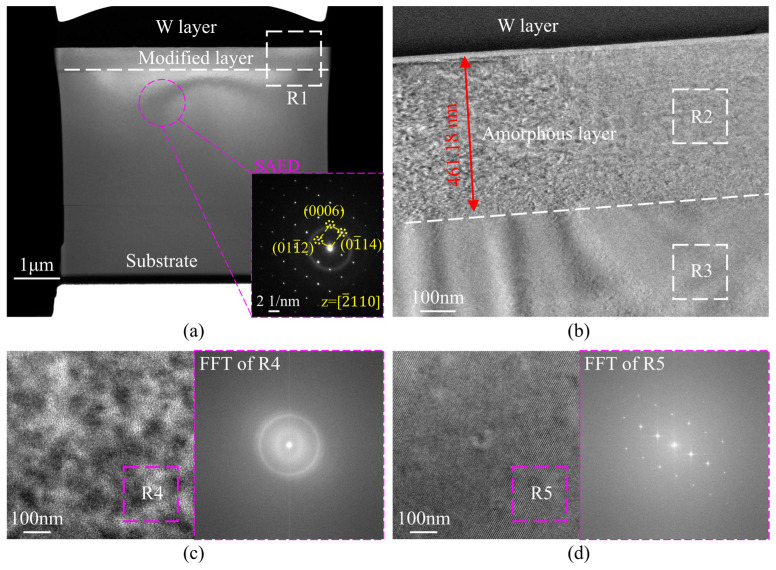
Microstructure of the modified layer in sapphire after phosphorus ion implantation. (**a**) Macroscopic morphology of the TEM sample; (**b**) magnified view of region R1; (**c**) local view of region R2; (**d**) local view of region R3.

The microstructural evolution mechanism of the modified layer was systematically characterized using TEM observation. Figure 14a illustrates the evolution of the subsurface microstructure. SAED patterns obtained along the [$\bar{2}$110] zone axis beneath the W protective layer (inset of Figure 14a) confirm the presence of crystalline phases. As shown in Figure 14b, a uniform modified layer with a thickness of 461.18 nm is observed beneath the protective layer. It should be noted that the measured modification depth is about 60 nm greater than the simulated result. This discrepancy arises because TRIM does not account for damage accumulation, which lowers the displacement energy. As the lattice becomes partially disordered, subsequent atoms are more easily displaced by incident ions, thereby intensifying the damage. Such dynamic changes in crystal integrity are beyond the capability of static TRIM simulations, resulting in an underestimation of damage formation. The fast Fourier transform (FFT) pattern of the R2 region (Figure 14c) exhibits characteristic diffuse ring features. As the observation area transitions from the shallow layer to the deeper bulk material, the FFT patterns display a regular matrix of diffraction spots (Figure 14d), consistent with the SAED pattern in the inset of Figure 14a, indicating that the portion of the modified layer near the bulk retains a high degree of crystallinity.

**Figure 15:**
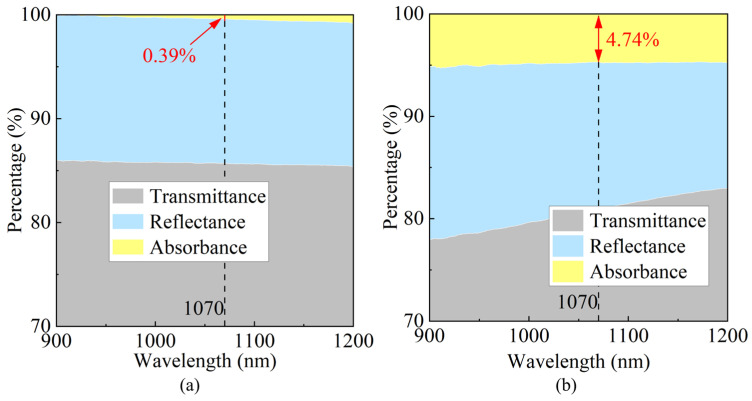
Optical properties of the C-plane sapphire crystal surface in the near-infrared region. (**a**) Before and (**b**) after modification.

The optical properties of sapphire samples before and after surface modification by ion implantation were measured in the near-infrared region (900–1200 nm) using a UV-Vis spectrophotometer (SolidSpec-3700, Shimadzu Corporation, Kyoto, Japan), including transmittance, reflectance, and absorbance, as shown in Figure 15. Taking the wavelength of 1070 nm as an example, the reflectance of the sample surface changed slightly from 14.05% to 14.27% after modification; the transmittance decreased from 85.56% to 80.99%, while the absorbance increased from 0.39% to 4.74%. Clearly, the decrease in transmittance is approximately consistent with the increase in absorbance. This phenomenon indicates that the microscopic defects introduced by ion implantation within the sapphire crystal enhance photon absorption in the near-infrared range, resulting in reduced transmitted light intensity.

### 3.3. Ductile–Brittle Transition Behavior

**Figure 16:**
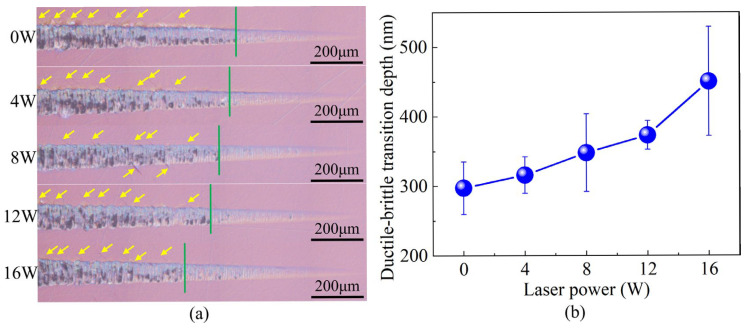
Analysis of surface morphology of modified sapphire after LADM. (**a**) Macroscopic morphology of the grooves where the yellow arrows indicate the locations of surface cracks; (**b**) effect of laser power on the DBTD.

Based on the finding that microscopic defects introduced by ion implantation enhance photon absorption in the near-infrared region of sapphire crystals, the effect of laser power on the machinability of modified sapphire was further investigated. Figure 16a presents the surface morphology of sapphire after LADM. Clearly, the micro-grooves generated along the cutting direction exhibit distinct brittle and ductile zones. At the initial stage of the groove, the surface is smooth and clean, with well-defined edges, indicating that the material removal mechanism is predominantly ductile. As the cutting depth increases, pits caused by brittle fracture gradually appear and their density continues to rise, eventually making brittle removal the dominant mechanism. The green solid lines in the figures mark the ductile–brittle transition locations, beyond which the groove surface is covered with cleavage patterns and severe tearing, characteristic of brittle defects as indicated by the yellow arrows. Moreover, with increasing laser power, the extent of the ductile region slightly expands, while the size and density of brittle fracture defects on the groove surface decrease marginally at higher powers. This effect can be attributed to the enhanced thermal softening at higher laser power, which improves the material’s plastic deformation capability, allowing chip formation to occur more easily through plastic flow.

Figure 16b compares the DBTD of modified sapphire under different laser powers. In conventional cutting (without laser assistance), the DBTD of the modified sapphire was 297.33 nm, which is consistent with previously reported experimental results [7,44,45,46]. With increasing laser power, the DBTD increased, reaching 450.67 nm at 16 W, representing a 51.57% improvement over conventional cutting. It is noteworthy that the DBTD during laser-assisted diamond machining of modified sapphire exhibits a relatively large standard deviation, indicating some instability in the cutting process. This is attributed to the fact that, although the absorption of the modified sapphire at the 1070 nm wavelength used for laser assistance is enhanced, the absolute value remains modest. Future work will focus on process optimization to further improve the machinability of the modified sapphire.

**Figure 17:**
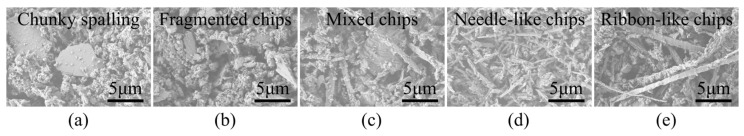
Chip morphology of ion-implanted sapphire after LADM at different laser powers. (**a**) 0 W; (**b**) 4 W; (**c**) 8 W; (**d**) 12 W; (**e**) 16 W.

To further investigate the evolution mechanism of material removal modes, Figure 17 shows the morphology of chips generated during LADM of modified sapphire at different laser powers. In conventional cutting (0 W, without in situ laser assistance), the chips primarily exhibit irregular fragments with micron-sized discontinuous block features of random shapes. Some areas display ripple patterns caused by stress waves, reflecting the spalling and fracture of the material during removal, as shown in Figure 17a. These observations indicate that during conventional cutting of implanted sapphire, the removal process is dominated by crack propagation and cleavage fracture. As illustrated in Figure 17b,c, with increasing laser power, the amount of blocky spalling in the chips gradually decreases. At 8 W, a small fraction of ribbon-like chips appears, indicating a transition from blocky fragments to mixed chips. With further power increase, the chip morphology evolves from needle-like to ribbon-like, as shown in Figure 17d,e, with almost no blocky spalling observed. These results further confirm that LADM can enhance the ductility of implanted sapphire.

## 4. Conclusions

This study investigated the cutting performance of laser-assisted diamond machining (LADM) of ion-implanted sapphire through simulation and experiments. Monte Carlo simulations were employed to examine the effects of implantation parameters, such as incidence angle and ion energy, on ion distribution, damage formation, and energy transfer, thereby elucidating the interaction mechanisms between high-energy ions and the target material. Phosphorus ions were implanted into sapphire crystals, and the microstructural evolution of the resulting modified layer was characterized using transmission electron microscopy (TEM) observation. Finally, groove cutting experiments were conducted to validate the improvement in machinability of the modified sapphire under LADM. The main conclusions drawn from the combined simulation and experimental results are as follows:

(1) The incidence angle and energy selection in the ion implantation process significantly affect the interaction mechanisms between ions and the target atoms. With increasing energy, the projected range of incident ions increases, the distributions of recoil atoms and energy deposition shift deeper into the material, and the damage evolves from a near-surface concentration to a more dispersed deep-layer profile.
(2) Under all energy conditions, at least 53% of the total energy of the incident ions is dissipated through vacancy formation, inducing internal defects within the crystal. Phonon-mediated energy dissipation serves as the primary pathway for converting kinetic energy into internal energy. Additionally, the total number of displacements equals the sum of vacancies and replacement collisions.
(3) Implantation of 300 keV phosphorus ions at a 7° incidence angle produced a uniform amorphous modified layer with a thickness of 461.18 nm. The resulting microstructural changes within the crystal increased the material’s absorption at 1070 nm from 0.39% to 4.74%, enabling the application of LADM techniques.
(4) LADM significantly enhances the ductile cutting performance of modified sapphire. With increasing laser power, the extent of the ductile region expands, while the size and density of brittle fracture defects on the groove surface decrease. At 16 W, the ductile–brittle transition depth increased to 450.67 nm, representing a 51.57% improvement over conventional cutting.

Overall, this study provides valuable insights into the interaction mechanisms between ions and the target material, as well as the material removal mechanisms during LADM of implanted sapphire. It also demonstrates the great potential of integrating ion implantation surface modification with ultraprecision machining to enhance the ductile cutting performance of extremely hard and brittle materials.

## Figures and Tables

**Table 1:** Parameters of groove cutting experiments.

**Description**	**Value**
Workpiece	C plane sapphire
Laser source	1070 nm continuous-wave laser
Tool nose radius	0.506 mm
Rake angle	−35°
Flank angle	10°
Cutting speed	200 mm/min
Groove slope	D/L = 1:1000
Laser power	0 W, 4 W, 8 W, 12 W, 16 W

## Data Availability

The original contributions presented in the study are included in the article, further inquiries can be directed to the corresponding author.
